# Mito-Genipin, a Novel Mitochondria-Targeted Genipin Derivative Modulates Oxidative Stress and Inflammation in Macrophages

**DOI:** 10.3390/antiox14111281

**Published:** 2025-10-25

**Authors:** Beatrice Angi, Daria Di Molfetta, Diana Pendin, Giuseppe Antoniazzi, Carlo Alberto Flora, Francesco De Leonardis, Martina Buono, Giuseppe Fiermonte, Ildiko Szabo, Andrea Mattarei, Tatiana Varanita

**Affiliations:** 1 Department of Biology, University of Padova, 35131 Padova, Italy; beatrice.angi@unipd.it (B.A.); carloalberto.flora@studenti.unipd.it (C.A.F.); martina.buono@studenti.unipd.it (M.B.); ildiko.szabo@unipd.it (I.S.); 2 Department of Biosciences, Biotechnologies and Environment, University of Bari, 70125 Bari, Italy; daria.dimolfetta@uniba.it (D.D.M.); francesco.deleonardis@uniba.it (F.D.L.); giuseppe.fiermonte@uniba.it (G.F.); 3 Neuroscience Institute, National Research Council (CNR), 35131 Padova, Italy; diana.pendin@cnr.it; 4 Department of Biomedical Sciences, University of Padua, 35131 Padova, Italy; 5 Department of Pharmaceutical and Pharmacological Sciences, University of Padova, 35131 Padova, Italy; giuseppe.antoniazzi@org.chem.ethz.ch

**Keywords:** genipin, mito-genipin, macrophages, ROS, UCP2

## Abstract

Genipin, a natural compound derived from *Gardenia jasminoides*, is widely used as an inhibitor of uncoupling protein 2 (UCP2), a protein located in the inner mitochondrial membrane (IMM) that plays a crucial role in regulating oxidative stress and cellular metabolism. Pharmacological inhibition of UCP2 has been explored as a strategy to modulate reactive oxygen species (ROS) and inflammatory responses. However, the utility of genipin is limited by its relatively low bioavailability and dose-dependent toxicity. To address these limitations, we developed mito-genipin, a mitochondria-targeted genipin derivative incorporating a triphenylphosphonium (TPP^+^) moiety, designed to enhance mitochondrial accumulation and thereby increase efficacy. In macrophages, mito-genipin induced mitochondrial hyperpolarization, elevated ROS production, and amplified pro-inflammatory cytokine expression compared with control or genipin treatment. In cells lacking UCP2, mito-genipin did not enhance ROS production. Our data identify mito-genipin as an effective modulator of oxidative stress and inflammation, supporting a putative link to UCP2 inhibition and highlighting potential implications in redox biology and immunomodulation.

## 1. Introduction

Genipin, a natural compound found in Gardenia jasminoides, selectively inhibits uncoupling protein 2 (UCP2)-mediated proton leak [1] across the mitochondrial inner membrane and has been used in traditional Chinese medicine for centuries in the context of treatment of diabetes, cancer, and inflammation. UCP2 (SLC25A8), along with the homologs UCP1 and UCP3, are mitochondrial carrier proteins showing different tissue expression and involved in physiological processes ranging from the regulation of insulin secretion to thermosensing and muscle fuel metabolism [2,3]. UCP2 is highly expressed in specific tissues such as cardiac tissue [4] pancreatic islets, in the lymphoid system, and in macrophages [5]. While UCP1 allows the re-entry of protons from the intermembrane space, thereby lowering the efficiency of oxidative phosphorylation and reducing the release of mitochondrial reactive oxygen species (ROS) production [3,6], the function of UCP2 and UCP3 in proton leak is less defined [3]. The currently accepted view is that UCP2 may possess dual transport function and facilitate both proton translocation and the export of C4 metabolites. In fact, our group has recently identified a metabolite (aspartate) transport function for UCP2 [7,8] and UCP3 [9]. At the same time, inhibition of UCP2 by genipin or reduction of its expression by siRNA has been reported to increase ROS production [7,10,11,12].

UCP2-deficient mice resisted toxoplasmosis via enhanced production of ROS from the macrophages in vivo [13] and genipin was shown to trigger ROS production also in RAW 264.7 macrophage cells [14]. In general, UCP2 was proposed to control immune cell activation by the production of mitochondrial ROS. UCP2 was demonstrated to directly affect the production of cytokines and therefore impact the outcome of infection, inflammation, and autoimmunity (for review, e.g., see [15]). Upon lipopolysaccharide (LPS) stimulation, deletion of UCP2 was shown to promote the expression of the inducible form of the NO synthase (iNOS), and to enhance ROS production as well as the release of pro-inflammatory cytokines in macrophages [16,17]. On the contrary, a reduced NO production was observed in LPS-stimulated UCP2-overexpressing macrophages [18].

Altogether, these studies clearly indicate a correlation between UCP2 expression, ROS production, and activation in macrophages. ROS can promote context-dependent polarization of macrophages to M1 pro-inflammatory or M2 anti-inflammatory states [19]. A recent study showed that genipin-mediated inhibition of UCP2 in alveolar macrophages increases mitochondrial ROS and selectively boosts mitochondrial ROS-dependent cytokine production upon bacterial challenge, highlighting that genipin can also amplify pro-inflammatory responses in alveolar macrophages [20]. In another study, genipin was shown to sustain polarization of M0 macrophages to the pro-regenerative M2 subtype when used as a crosslinker in biomaterials [21]. On the other hand, reduction of intracellular ROS levels restored mitochondrial function and repolarized M1 macrophages into M2 phenotypes [22].

Given the pivotal role of macrophages and their activation and polarization in various pathophysiological contexts, ranging from cancer to infection, inflammatory responses, and tissue repair, we aimed to efficiently modulate UCP2 activity in macrophages.

As mentioned above, UCP2 is located in the IMM; therefore, the action of genipin may be limited by slow and incomplete diffusion of the molecule to the mitochondria. Thus, we exploited the strategy previously used by our groups with success for different mitochondrial ion channels, such as mitoKv1.3 [23], mitoTASK-3 [24] and mitoKCa3.1 [25]. In particular, we fused genipin to the positively charged TPP^+^ molecule, which promotes the accumulation of the drug in mitochondria, as they exhibit a highly negative membrane potential on the matrix side [26].

Here, we show, using murine macrophages, that mito-genipin is able to recapitulate the main characteristics of UCP2 KO macrophages regarding ROS and cytokine production, while being more effective than genipin at promoting pro-inflammatory features.

## 2. Materials and Methods

### 2.1. Chemistry, Materials, and Instruments

Reagents and solvents were purchased from Sigma-Aldrich (Milan, Italy) and used as received. Flash column chromatography was performed on silica gel (Macherey-Nagel 60, 230–400 mesh granulometry (0.063–0.040 mm)) under nitrogen pressure. ^1^H and ^13^C NMR spectra were recorded with a Bruker 400 Avance III HD operating at 400 MHz (for ^1^H NMR) and 101 MHz (for ^13^C NMR). Chemical shifts (δ) are given in parts per million (ppm) relative to the signal of the solvent. The following abbreviations were used to indicate multiplicities: s, singlet; d, doublet; dd, doublet of doublets; t, triplet; m, multiplet; br, broad signal. ESI-MS analysis was performed using an Agilent Technologies 1100 Series system. The ESI source operated in full-scan positive ion mode, using the following ESI parameters: nebulizer pressure, 20 psi; dry gas flow, 5 L/min; and dry gas temperature, 325 °C. The flow rate was 0.05 mL/min. HPLC-UV/ESI-MS analysis of the final product was performed using an Agilent Technologies 1100 Series system, equipped with a reversed-phase column (Kinetex C18, 5 µm, 150 × 4.6 mm, 100 Å, Phenomenex) and a flow rate of 1.0 mL/min. Solvents A and B were water +0.1% formic acid and CH_3_CN +0.1% formic acid, respectively. The gradient for B was as follows: 1 min at 10%, then from 10% to 100% in 25 min. The eluate was monitored at 250 nm. HPLC-MS analysis was performed using an ESI source operating in full-scan positive ion mode, with the following ESI parameters: nebulizer pressure, 50 psi; dry gas flow, 10 L/min; and dry gas temperature, 350 °C.

*methyl 1-((tert-butyldimethylsilyl)oxy)-7-(((tert-butyldimethylsilyl)oxy)methyl)-1,4a,5,7a-tetrahydrocyclopenta[c]pyran-4-carboxylate* (1). To a solution of genipin (2.21 mmol, 1.0 eq) and AgNO_3_ (6.63 mmol, 3.0 eq) in DMF (3.5 mL) was added a solution of *t*-butyldimethylsilyl chloride (6.63 mmol, 3.0 eq) in DMF (2.0 mL), and the reaction mixture was stirred for 14 h at room temperature. Then, the reaction was quenched with sat. NaHCO_3_ (50 mL) and extracted with EtOAc (3 × 50 mL). The combined organic phases were washed with brine (3 × 50 mL), dried over anhydrous MgSO_4_, and the solvent was removed under vacuum. The crude product was purified by flash chromatography (eluent CHCl_3_/EtOAc, 98:2) to obtain the pure product (1.95 mmol, 88% yield). ^1^H NMR (400 MHz, CDCl_3_) δ 7.48 (d, *J* = 0.6 Hz, 1H), 5.81 (s, 1H), 4.84 (d, *J* = 7.7 Hz, 1H), 4.35 (d, *J* = 14.8 Hz, 1H), 4.21 (dd, *J* = 14.8, 2.1 Hz, 1H), 3.72 (s, 3H), 3.18 (q, *J* = 7.7 Hz, 1H), 2.89–2.78 (m, 1H), 2.46 (t, *J* = 7.5 Hz, 1H), 2.10–2.00 (m, 1H), 0.91 (d, *J* = 2.9 Hz, 18H), 0.13 (d, *J* = 7.1 Hz, 6H), 0.06 (d, *J* = 3.2 Hz, 6H). ^13^C NMR (101 MHz, CDCl_3_) δ 168.05, 152.33, 143.71, 126.62, 110.94, 96.83, 62.00, 51.15, 48.49, 38.66, 36.14, 25.90, 25.72, 18.35, 17.91, −4.17, −4.96, −5.30, −5.35. ESI^+^(MS): *m*/*z*: 455 [M+H]^+^.

*methyl 1-((tert-butyldimethylsilyl)oxy)-7-(hydroxymethyl)-1,4a,5,7a-tetrahydrocyclopenta[c]pyran-4-carboxylate* (2). A solution of 1 (1.91 mmol, 1.0 eq) and PPTS (1.91 mmol, 1.0 eq) in EtOH (10 mL) was stirred at room temperature for 2 days. After solvent removal under vacuum, the crude product was purified by flash chromatography (eluents: a gradient of petroleum ether/Et2O from 5:1 to 1:4) to obtain 2 as a white powder (1.78 mmol, 93% yield). ^1^H NMR (400 MHz, CDCl_3_) δ 7.50 (s, 1H), 5.83 (s, 1H), 4.82 (d, *J* = 8.1 Hz, 1H), 4.29 (s, 2H), 3.73 (s, 3H), 3.18 (q, *J* = 8.4 Hz, 1H), 2.92–2.82 (m, 1H), 2.56 (t, *J* = 7.9 Hz, 1H), 2.12–2.02 (m, 1H), 1.98 (t, *J* = 6.1 Hz, 1H), 0.92 (s, 9H), 0.15 (d, *J* = 9.1 Hz, 6H). ^13^C NMR (101 MHz, CDCl_3_) δ 167.90, 152.29, 143.56, 128.19, 110.96, 97.06, 61.52, 51.24, 48.47, 38.94, 36.30, 25.72, 17.94, −4.19, −4.90. ESI^+^(MS): *m*/*z*: 341 [M+H]^+^.

*methyl 1-((tert-butyldimethylsilyl)oxy)-7-(((5-chloropentanoyl)oxy) methyl)-1,4a,5,7a-tetrahydrocyclopenta[c]pyran-4-carboxylate* (3). A solution of 2 (0.862 mmol, 1.0 eq), Et_3_N (1.72 mmol, 2.0 eq), and DMAP (0.431 mmol, 0.5 eq) in CH_2_Cl_2_ (6 mL) was cooled to 0 °C. Then, 5-chlorovaleroyl chloride (1.293 mmol, 1.5 eq) was added dropwise, and the reaction was stirred overnight at room temperature. The reaction was quenched with brine (50 mL) and HCl 0.5 M (50 mL) and extracted in CH_2_Cl_2_ (3 × 50 mL). The crude product was purified by silica gel chromatography (eluent Petroleum ether/Et_2_O, 5:1) to obtain 0.804 mmol of 3 (93% yield). ^1^H NMR (400 MHz, CDCl_3_) δ 7.50 (s, 1H), 5.86 (s, 1H), 4.84–4.66 (m, 3H), 3.72 (s, 3H), 3.55 (t, *J* = 6.0 Hz, 2H), 3.19 (q, *J* = 8.3 Hz, 1H), 2.87 (dd, *J* = 16.6, 8.5 Hz, 1H), 2.56 (t, *J* = 7.3 Hz, 1H), 2.38 (t, *J* = 6.8 Hz, 2H), 2.12–2.02 (m, 1H), 1.80 (dd, *J* = 8.1, 5.1 Hz, 4H), 0.91 (s, 9H), 0.13 (d, *J* = 8.0 Hz, 6H). ^13^C NMR (101 MHz, CDCl_3_) δ 172.71, 167.90, 152.48, 138.57, 130.42, 110.73, 96.79, 62.68, 51.23, 48.35, 44.43, 38.94, 35.97, 33.36, 31.85, 25.67, 22.27, 17.88, −4.27, −5.01. ESI^+^(MS): *m*/*z*: 459 [M+H]^+^.

*methyl 1-((tert-butyldimethylsilyl)oxy)-7-(((5-iodopentanoyl)oxy)methyl)-1,4a,5,7a-tetrahydrocyclopenta[c]pyran-4-carboxylate* (4). Compound 3 (0.527 mmol, 1.0 eq) was dissolved under nitrogen in anhydrous acetone (5 mL) saturated with NaI. The suspension was maintained at 70 °C under stirring and in the dark overnight. After the removal of the solvent under vacuum, ethyl acetate (100 mL) was added, and the mixture was washed with water (4 × 75 mL). The combined aqueous layers were extracted with dichloromethane (2 × 50 mL). The combined organic layers were dried over MgSO_4_, filtered, and the solvent was removed under reduced pressure. The crude product was purified by flash chromatography (eluent EtOAc/petroleum ether, 6:4) as eluent to afford compound 4 (97% yield). ^1^H NMR (400 MHz, CDCl_3_) δ 7.50 (s, 1H), 5.86 (s, 1H), 4.85–4.64 (m, 3H), 3.72 (s, 3H), 3.20 (m, *J* = 6.9 Hz, 3H), 2.88 (dd, *J* = 16.6, 8.5 Hz, 1H), 2.56 (t, *J* = 7.8 Hz, 1H), 2.37 (t, *J* = 7.2 Hz, 2H), 2.12–2.04 (m, 1H), 1.81 (dp, *J* = 22.5, 7.0 Hz, 4H), 0.92 (s, 9H), 0.14 (d, *J* = 8.0 Hz, 6H). ^13^C NMR (101 MHz, CDCl_3_) δ 172.65, 167.90, 152.48, 138.56, 130.45, 110.73, 96.79, 62.69, 51.22, 48.36, 38.95, 35.97, 33.03, 32.73, 25.82, 25.68, 17.89, 5.74, −4.25, −4.99. ESI^+^(MS): *m*/*z*: 551 [M+H]^+^.

*(5-((1-hydroxy-4-(methoxycarbonyl)-1,4a,5,7a-tetrahydrocyclopenta[c]pyran-7-yl)methoxy)-5-oxopentyl)triphenylphosphonium iodide* (mito-genipin). To a solution of compound 5 (0.0616 mmol, 1.0 eq.) dissolved in THF/ACN 1:1 (0.8 mL), was added acetic acid (1.1 eq) and KF (10.0 eq), and the reaction mixture was stirred overnight. After the removal of the solvent, the crude product was purified by silica gel chromatography (eluent chloroform/Acetone, 6:4) to yield *mito*-genipin as a white powder (yield 70%, purity > 95% by UPLC analysis). ^1^H NMR (400 MHz, CDCl_3_) δ 7.87–7.69 (m, 15H), 7.53 (s, 1H), 5.94 (d, *J* = 12.7 Hz, 1H), 5.02–4.63 (m, 3H), 3.85–3.68 (m, 5H), 3.18 (dd, *J* = 16.9, 8.5 Hz, 1H), 2.88 (m, *J* = 16.4, 8.1 Hz, 1H), 2.71–2.65 (m, 1H), 2.58 (t, *J* = 6.8 Hz, 2H), 2.09–1.99 (m, 3H), 1.81–1.72 (m, 3H). ^13^C NMR (101 MHz, CDCl_3_) δ 173.39, 168.13, 153.25, 138.52, 135.28 (d, J = 3.1 Hz), 133.75 (d, J = 10.1 Hz), 132.49, 130.68 (d, J = 12.6 Hz), 118.02 (d, J = 86.2 Hz), 110.06, 95.99, 63.12, 51.23, 47.37, 39.00, 36.06, 33.50, 25.47 (d, J = 17.1 Hz), 22.88 (d, J = 51.1 Hz), 21.88 (d, J = 4.3 Hz). ESI^+^(MS): *m*/*z*: 571 [M-I]^+^.

#### 2.1.1. Cell Cultures

In vitro experiments were conducted using primary mouse bone marrow-derived macrophages (BMDMs) and the murine macrophage cell line RAW 264.7 (ATCC-SC-6003). BMDMs were prepared as in [27]. Briefly: bone marrow cells from the femur and tibia of 10-wk-old wild-type C57Bl6/J (Charles River) were cultured in Dulbecco’s modified Eagle medium (DMEM) containing 10% heat-inactivated FBS, in the presence of M-CSF (Miltenyi Biotec, Cologne, Germany), 25 ng/mL in not-treated tissue culture plates. Differentiation into macrophages was analyzed using the BD LRS Fortessa X20 flow cytometer after staining cells for 10 min at room temperature in the dark with the macrophage-specific marker, anti-F4/80 (Thermo Fisher 47-4801-82) antibodies. On day 7, differentiated macrophages (BMDMs) were detached from the plate using cold phosphate-buffered saline (PBS) + 1 mM ethylenediaminetetraacetic acid (EDTA) and used on the same day. RAW 264.7 were cultured in Dulbecco’s modified Eagle medium (DMEM, Invitrogen) supplemented with 10% fetal bovine serum (FBS, BioSpa S.p.A.), 100 U/mL penicillin, and 100 U/mL streptomycin (Life Technologies). All cells were maintained at 37 °C and 5% CO_2_.

#### 2.1.2. Generation of UCP2 Knockout iPSC

hiPSCs were fibroblast-derived BYS0112 (ACS-1026™) obtained from ATCC. Cells were cultured under standard conditions and subjected to CRISPR/Cas9-mediated genome editing to generate the UCP2 knockout line. A single guide RNA (sgRNA) sequence (ACTGTTTGACAGAATCATAC) targeting the coding region of UCP2 (nt 297–316) on the reverse strand was cloned into the pSpCas9(BB)-2A-GFP (PX458) plasmid (Addgene #48138) [28,29]. The verified construct was transfected into iPSCs using Lipofectamine STEM Transfection Reagent (ThermoFisher, Thermofisher, Monza, Italy, #STEM00001). Two days post-transfection, single GFP-positive cells were sorted and expanded. The target genomic region was amplified by PCR using flanking primers and sequenced. A full UCP2 knockout clone was successfully obtained, carrying a 1-nt deletion on one allele and a 10-nt deletion on the other, both resulting in frameshift mutations.

#### 2.1.3. Animals

8–9-week-old C57Bl/6J mice acquired from Jackson Laboratories were housed for one week in the Department of Biology (Padova) facility before being used for organ collection, following procedures approved by the University of Padova Ethical Committee for Animal Welfare (OPBA) and by the Italian Ministry of Health (Permit Number 144/2022-PR), in compliance with Italian Law DL 26/2014, embodying UE Directive 2010/63/EU.

#### 2.1.4. Cell Death Analysis

Cells were treated with various concentrations of Genipin and Mito-genipin (0, 10, 20, 40, 80, 160, 320 μM) for 24 h, and cell viability was assessed using MTS assay (G3581, Promega, Promega, Milan, Italy), following the manufacturer’s instructions. Cell viability was measured as the percentage of control (DMSO 0.1%). Alternatively, cell death was assessed using the Annexin-V Apoptosis detection kit (Invitrogen, Cat. #BMS500FI) following the manufacturer’s instructions. Annexin V-FITC and PI staining were detected by flow cytometry using a LSRFortessa X-20 (BD Biosciences). Analysis was performed on single-cell gated populations. Populations were defined as dead/necrotic (PI^+^ Annexin V^−^), late apoptotic (PI^+^ Annexin V^+^), early apoptotic (PI^−^ Annexin V^+^), and viable (PI^−^ Annexin V^−^). Cell viability was measured as the percentage of Annexin-V and PI-negative events.

#### 2.1.5. MitoSOX Assay

BMDMs, RAW 264.7 and Human iPSCs were incubated with 5 µM MitoSOX (Thermofisher, M36008) and 1 µM Cyclosporine H (Merck, SML1575) for 30 min at 37 °C in HBSS supplemented with 10 mM HEPES buffer (pH 7.4). Finally, the stained cells were treated with 10 µM, 20 µM, and 40 µM, mito-genipin or genipin, 10 µM antimycin A for 30 min, while the control group received 0.1% DMSO (Sigma-Aldrich, D2650). Data were analyzed using the BD LRS Fortessa X20 flow cytometer. At least 10,000 events per condition were acquired, and results were expressed as mean fluorescence intensity (MFI).

#### 2.1.6. TMRM Assay

BMDMs and RAW 264.7 cells were incubated with 20 nM TMRM (Thermofisher, 134361) and 1 µM Cyclosporine H for 30 min at 37 °C in HBSS supplemented with 10 mM HEPES buffer (pH 7.4). Stained cells were treated with either 10 µM or 20 µM mito-genipin or genipin, 2 µM FCCP, 2 µM Oligomycin, and analyzed using a S3e^TM^ Cell Sorter (BioRad) after 60 s of treatment. The control groups received no treatment (DMSO), 2 µM FCCP or 2 µM Oligomycin. For kinetic analysis of mitochondrial membrane potential, fluorescence changes were monitored for 60 min, with measurements taken every 5 min using a BioTek Synergy H1 (Agilent) multimode plate reader. Changes in TMRM fluorescence intensity over time were used as an indicator of mitochondrial polarization dynamics and expressed as a percentage of the initial control value.

#### 2.1.7. Extracellular Flux Assay

Agilent Seahorse XF Pro analyzer in a 96-well format was used to measure oxygen consumption rate (OCR) and extracellular acidification rate (ECAR). RAW 264.7 cells were seeded at 6 × 10^4^ cell/well in 50 μL of DMEM (Sigma-Aldrich D5030) supplemented with 25 mM glucose, 10 mM sodium pyruvate, and 2 mM L-glutamine on an Agilent Seahorse XF96 cell culture. Before the assay, the plate was placed in a 37 °C incubator without CO_2_ for 25–30 min to ensure the cells were completely attached. OCR was measured at preset time intervals upon the preprogrammed additions of 20 μM genipin or mito-genipin and the following modulators of respiration: 2 μM oligomycin (Merk O4876), 2 μM fluorocarbonyl cyanide phenylhydrazone (FCCP Merk 2920), and 1 μM rotenone + 1 μM antimycin A (Merk A8674).

#### 2.1.8. Macrophage Activation

RAW 264.7 cells were seeded in 6-well plates at a density of 5 × 10^5^ cells per well, and cultured in DMEM medium supplemented with 10% FBS, 1% penicillin, and streptomycin, and maintained at 37 °C and 5% CO_2_. After 24 h, the medium was gently aspirated, and fresh medium with activating stimuli was added [30] was added for an additional 24 h. Classically activated macrophages were obtained by polarizing cells with a combination of 100 ng/mL LPS (5970-44-01, Invivogen) and 100 ng/mL IFN-γ (130-105-774, Miltenyi Biotec) in the presence or absence of 10 µM, 20 µM, and 40 µM, mito-genipin or genipin. Alternatively, activated macrophages were polarized with 20 ng/mL of IL-4 (130-097-761, Miltenyi Biotec) and 20 ng/mL IL-13 (130-094-070, Miltenyi Biotec) in the presence or absence of 10 µM, 20 µM, and 40 µM, *mito*-genipin or genipin.

### 2.2. Fluorescence and Confocal Imaging

RAW 264.7 macrophages were seeded onto 18 mm diameter glass coverslips, and fluorescence and confocal imaging were performed after 24 h at 80–90% cell confluence. During the experiment, each coverslip was placed into an open-topped chamber, and cells were maintained in an extracellular-like medium (EC), i.e., a modified Krebs–Ringer buffer containing (in mM) 135 NaCl, 5 KCl, 1 MgCl_2_, 20 HEPES, 11 glucose, pH 7.4 at 37 °C. In live imaging experiments, fluorescence was recorded using a 40× objective (SFluor 40× N.A. 1.3, Nikon, Campi Bisenzio, Italy) on an inverted microscope (Nikon Ti-E). Fluorescence illumination at 555 nm was obtained using a monochromator (Optoscan, CAIRN-Research) controlled by NIS-ELEMENTS AR (Nikon) software (Version 4.5). The emitted fluorescence was collected using an FF-570-Di01 Dichroic and a 620/52 nm filter (Semrock). Images were acquired upon 500 ms exposure, with binning 2 × 2, by a Zyla-CMOS 4.2-P camera (Andor, Oxford Instruments). After the first acquisition, genipin or mito-genipin (20 μM) were added, then images were acquired every 60 s for 1 h. Regions of interest (ROIs), corresponding to the cells and to the coverslip background were selected on the images collected. The background values were subtracted and ROIs fluorescence at each acquisition (F) normalized to the fluorescence before addition of the substances (F_0_) was plotted as F/F_0_ over time. For confocal imaging, genipin or mito-genipin at a concentration of 20 μM were preincubated for 2h at 37 °C in EC. Then, MitoTracker Deep Red (50 nM, Thermo Fisher #M22426) was added before imaging. Confocal analysis was performed on a Leica Stellaris 8 microscope equipped with a 63×/1.4 N.A. Plan Apochromat objective, upon excitation by a WLL set at 580 nm and 642 nm. Images were acquired with HyD detectors, bidirectional scanner, zoom 2, image size 1024 × 1024 pixels.

### 2.3. Gene Expression Analysis

RNA was extracted using a RNeasy mini kit (74106, Qiagen). cDNA was obtained using the SuperScript IV Reverse Transcriptase (Thermofisher, #18091050) following the manufacturer’s instructions. For each reaction, 2 μg of RNA was reverse-transcribed according to the manufacturer’s instructions. The resulting cDNA was then diluted and amplified with qRT-PCR using SYBR Green PCR Master Mix (Thermofisher, #4309155). Actin was used as a housekeeping control. CT values were first normalized to the housekeeping genes (ΔCT) and then compared to the control sample (ΔΔCT). This relative normalized expression is shown in the figures. The primer sequences for the mouse gene used in RT-qPCR analyses were as follows: *Ucp2* forward 5′-TCATCACTTTCCCTCTGGATACC-3′ and *Ucp2* reverse 5′-GCGCACTAGCCCTTGACTCT-3′; *Tnf-α* forward 5′-ATGAGCACAGAAAGCATGA-3′ and *Tnf-α* reverse 5′-AGTAGACAGAAGAGCGTGGT-3′; *Il-6* forward 5′-CAAAGCCAGAGTCCTTCAGAG-3′ and *Il-6* reverse 5′-TGGTCCTTAGCCACTCCTTC-3′; *Nos2* forward 5′-GTTCTCAGCCCAACAATACAAGA-3′ and *Nos2* reverse 5′- GTGGACGGGTCGATGTCAC-3′; *Arg1* forward 5′- CGTAGACCCTGGGGAACACTAT-3′ and *Arg1* reverse 5′- TCCATCACCTTGCCAATCCC-3′; *Mrc1* forward 5′-CAACCAAAGCTGACCAAAGG-3′ and *Mrc1* reverse 5′- CCGGCACCTATCACAATCAG -3′. 

### 2.4. Protein Extraction and Western Blotting

Cells were lysed on ice using RIPA buffer (50 mM Tris, 150 mM NaCl, 1% sodium deoxycholate, 1 mM EDTA, 1% Triton X-100, 0.1% SDS, pH 7.4) freshly supplemented with a protease inhibitor cocktail (Sigma, P8340). Lysates were collected by scraping, sonicated twice for 3 s with 10 s pauses on ice, incubated for 30 min, and centrifuged at 1000× *g* for 10 min at 4 °C. Supernatants were aliquoted and stored at −80 °C. Protein concentrations were determined using the Pierce BCA Protein Assay Kit (Thermo Scientific). For SDS-PAGE, protein samples (15 µg per lane) were mixed with 4× Laemmli loading buffer (Tris, SDS, glycerol, 2-mercaptoethanol, and bromophenol blue), boiled at 95 °C for 10 min, briefly centrifuged, and loaded onto polyacrylamide gels. After electrophoresis, proteins were transferred to nitrocellulose membranes, blocked, and probed with a primary antibody against UCP2 (diluted 1:1000), kindly provided by Elena Pohl [29]. Immunoreactive bands were detected by chemiluminescence using an HRP-conjugated anti-rabbit secondary antibody and the SuperSignal West Pico PLUS Chemiluminescent Substrate (Thermo Scientific).

### 2.5. Reconstitution of Recombinant UCP2 into Liposomes and Transport Measurements

Human UCP2 was expressed as inclusion bodies (IBs) in *E. coli* C0214 (*DE3*), then solubilized and reconstituted into liposomes as previously described [8,31]. During reconstitution, proteoliposomes were preloaded with 20 mM aspartate. Following the removal of the external substrate by gel filtration chromatography, transport was initiated by adding 1 mM [^14^C]-aspartate, together with various concentrations (0.05–1 mM) of mito-genipin or genipin dissolved in DMSO. In control experiments, DMSO was added alone. Transport was terminated after 1 min by adding 10 mM pyridoxal-5′-phosphate (PLP) and 10 mM bathophenanthroline (BAT) according to the “inhibitor-stop” method [7,8]. In blank samples, the inhibitors were added at the beginning together with the radiolabeled substrate. Finally, the external substrate was removed, and the radioactivity retained inside the liposomes was measured. Experimental values were corrected by subtracting the corresponding blank values.

## 3. Results

### 3.1. Synthesis of the Mitochondriotropic Analogue of Genipin, Mito-Genipin

**Figure 1:**
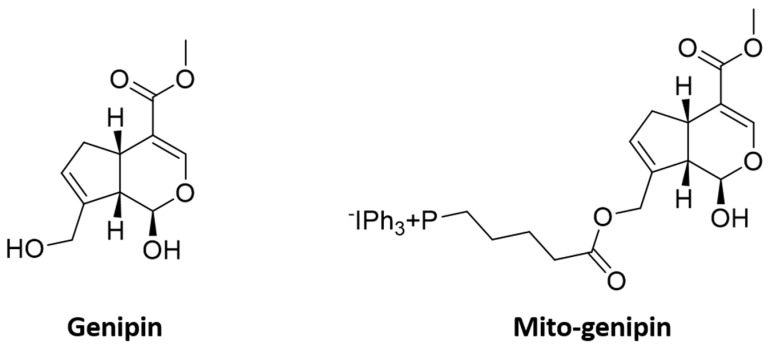
Molecular structures of the mitochondrial UCP2 inhibitor genipin and of the mitochondriotropic analogue mito-genipin synthesized in this work.

Here, we report the synthesis of a novel mitochondria-targeted derivative of genipin, an organic compound known for its ability to inhibit UCP2. The selective mitochondrial targeting was achieved through covalent conjugation of genipin to a TPP^+^ lipophilic cation (Figure 1).

**Scheme 1:**
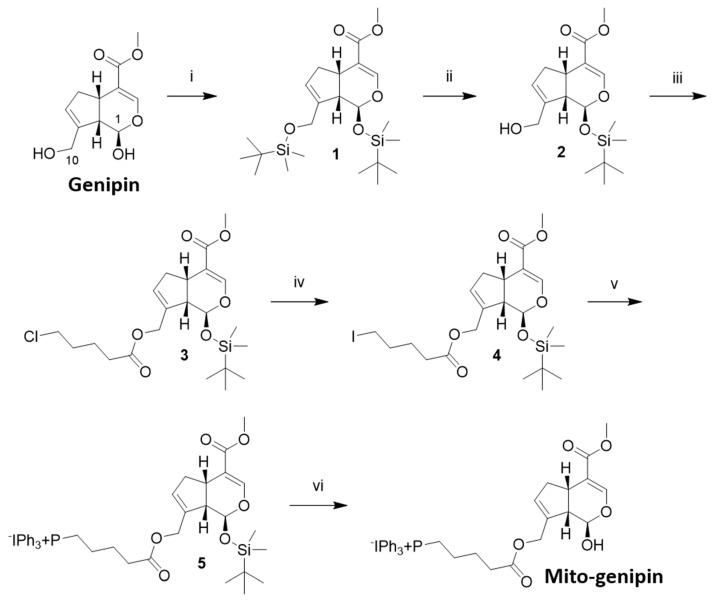
Synthesis of mito-genipin. Reagents and conditions: (i) TBDMS-Cl, AgNO_3_, DMF; (ii) PPTS, EtOH; (iii) 5-chlorovaleroyl chloride, Et_3_N, DMAP, CH_2_Cl_2_; (iv) NaI, Acetone; (v) PPh_3,_ neat; (vi) AcOH, KF, THF/ACN 1:1 HPLC-UV/ESI-MS analysis of the final product and physico-chemical characterization was performed as described in detail in the materials and methods section.

TPP^+^ groups are widely used to direct small organic pharmaceuticals to mitochondria, exploiting the negative potential (ΔΨm −180 mV) across the IMM [32]. In designing mito-genipin, we strategically conjugated the TPP^+^ group via a valeroyl ester spacer at the C10 position (10-OH) of the natural compound genipin. This design was based on previous research [33] showing that substitution at the 10-OH position of genipin is well-tolerated and preserves the pharmacological properties of the parent compound. The synthesis of mito-genipin is illustrated in Scheme 1. It was achieved starting from genipin, by silylation of the free hydroxyl groups (at positions C1 and C10) with t-butyldimethylsilyl chloride (i in Scheme 1), followed by selective de-silylation of the 10-OH group, promoted by a catalytic amount of PPTS in ethanol (ii in Scheme 1). Reaction of the free 10-OH group with 5-chloro-valeroylchloride (iii in Scheme 1), followed by exchange of chlorine with iodine, and iodine with TPP^+^ (iv–v in Scheme 1) led, after the cleavage of the second silyl protecting group (vi in Scheme 1), to the desired mito-genipin in good overall yield and excellent purity.

### 3.2. Assessment of Mito-Genipin Cytotoxicity in Macrophages

**Figure 2:**
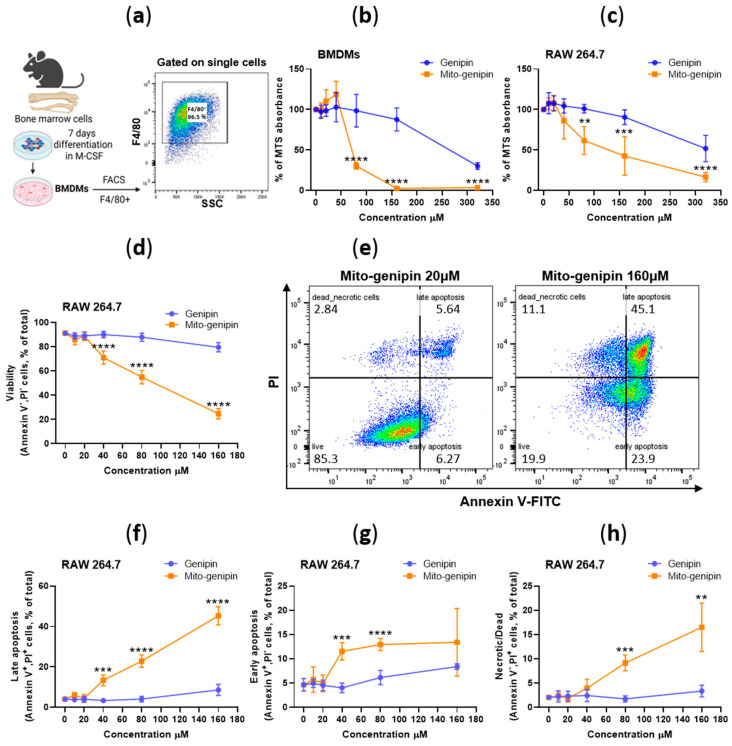
Effect of mito-genipin on cell viability in BMDMs and RAW 264.7 Macrophages. (**a**) Scheme showing preparation and differentiation of murine bone marrow-derived macrophages (BMDMs). Cell viability assessed by MTS in BMDMs (**b**) and RAW 264.7 cells (**c**) treated with increasing concentrations (0–320 µM) of mito-genipin or genipin for 24 h. Data points represent means ± SD of five independent experiments for each cell type. (**d**) Viability of RAW 264.7 cells treated with increasing concentrations of mito-genipin or genipin for 24 h, measured by Annexin V/PI staining. (**e**) Representative dot plots showing viability and cell death profiles of RAW 264.7 cells treated with 20 μM or 160 μM mito-genipin. Populations are defined as dead/necrotic (PI^+^ Annexin V^−^), late apoptotic (PI^+^ Annexin V^+^), early apoptotic (PI^−^ Annexin V^+^), and viable (PI^−^ Annexin V^−^). Analysis was performed on single-cell gated populations. Percentage of late apoptotic (**f**), early apoptotic (**g**), and necrotic (**h**) RAW 264.7 cells after treatment with increasing concentrations of mito-genipin or genipin for 24 h. Data points represent means ± SD of four independent experiments. ** *p* < 0.01; *** *p* < 0.001; **** *p* < 0.0001 indicate statistical significance relative to untreated controls in an unpaired Student’s *t*-test.

To investigate the cytotoxic effects of mito-genipin in macrophages, we first generated primary bone-marrow-derived macrophages (BMDMs) from mice (Figure 2a) and exposed them to increasing concentrations (0–320 µM) of the drug or its parent compound, genipin, for 24 h (Figure 2b), illustrating that mito-genipin is more efficient in reducing cell viability compared to genipin. To further validate our findings and investigate potential differences between primary and immortalized macrophages, we also conducted parallel experiments using the RAW 264.7 cell line (Figure 2c). Cell viability, assessed by MTS assay, revealed comparable dose-dependent reductions in both cell types following mito-genipin treatment. Significant decreases in viability were observed at concentrations of 80 µM and above in both BMDMs and RAW 264.7 cells. BMDMs and RAW 264.7 cells showed similar sensitivity to mito-genipin, with only slight differences at higher concentrations. In both cell types, mito-genipin induced significant cytotoxicity at a concentration of around 80 µM, with viability sharply declining at 160 µM and 320 µM. The estimated EC50 was slightly below 80 µM in BMDMs and approximately 80 µM in RAW 264.7 cells. In contrast, genipin induced weaker effects in both cell types, with significant cytotoxicity only observed at the highest concentrations tested, consistent with previous reports [21].

To further characterize the nature of mito-genipin-induced cell death, we focused on RAW 264.7 cells. We performed Annexin V/PI staining in these cells (Figure 2d–h). Flow cytometry analysis revealed that mito-genipin triggers significant apoptotic and, to less extent, necrotic cell death at concentrations above 40 µM. These results corroborate the MTS assay findings and provide additional insight into the mechanism of mito-genipin-induced cell death.

Based on these findings, we selected concentrations up to 40 µM for subsequent experiments to evaluate the effects of mito-genipin on macrophage function independent of cytotoxicity.

### 3.3. Mito-Genipin Efficiently Localizes to Mitochondria

**Figure 3:**
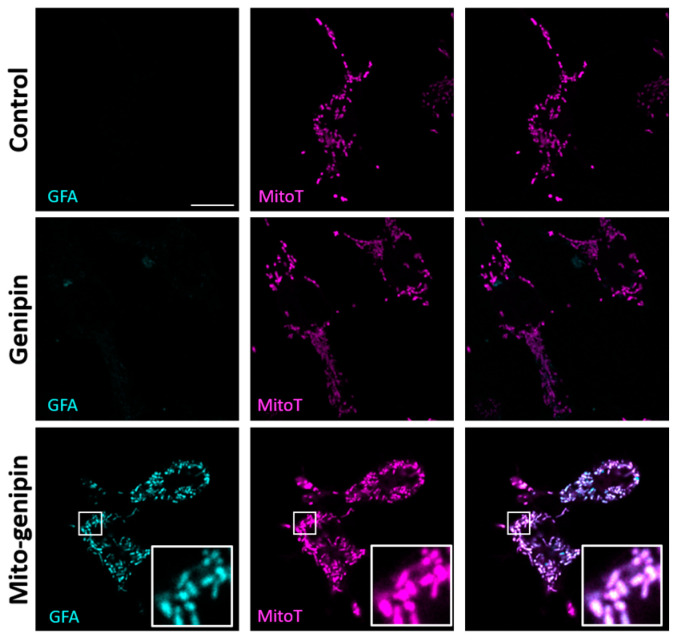
Mito-genipin accumulation in mitochondria of RAW 264.7 cells. Representative confocal images of RAW 264.7 cells stained with MitoTracker Deep Red (MitoT, magenta) and incubated with genipin, mito-genipin, or vehicle as control for 2 h. Genipin fluorescent adducts (GFA), whose formation is efficiently detectable after 2 h (See Figure S1) are shown in cyan. Merged images of the two fluorescence channels are shown in the rightmost column. Scale bar, 10 μm.

To provide a direct readout of mitochondrial accumulation and validate our design strategy, we aimed at assessing the proper mitochondrial accumulation of mito-genipin. We thus exploited the inherent capability of genipin to form fluorescent adducts upon reaction with primary amine residues in situ [34]. Although native genipin is non-fluorescent, its covalent coupling to protein amines yields a conjugated fluorophore (e.g., via crosslink reaction) that can be visualized upon excitation in the visible region [35]. Thus, RAW 264.7 macrophages were incubated with either mito-genipin or native genipin and subsequently imaged by confocal microscopy using excitation at 575 nm, collecting emission between 585 and 640 nm, in accordance with previous reports [36,37]. The mitochondriotropic derivative produced a distinct fluorescence pattern with morphology consistent with mitochondria, whereas the control genipin yielded only a weak and diffuse fluorescence. Colocalization experiments with mitochondria-labelling dye MitoTracker resulted in a substantial overlap between the two fluorescence channels (Figure 3). These findings demonstrate that the mitochondriotropic modification enabled efficient targeting to the organelle, where mito-genipin formed a detectable fraction of stable fluorescent adducts with local proteins.

### 3.4. Mito-Genipin Induces Mitochondrial Dysfunction

**Figure 4:**
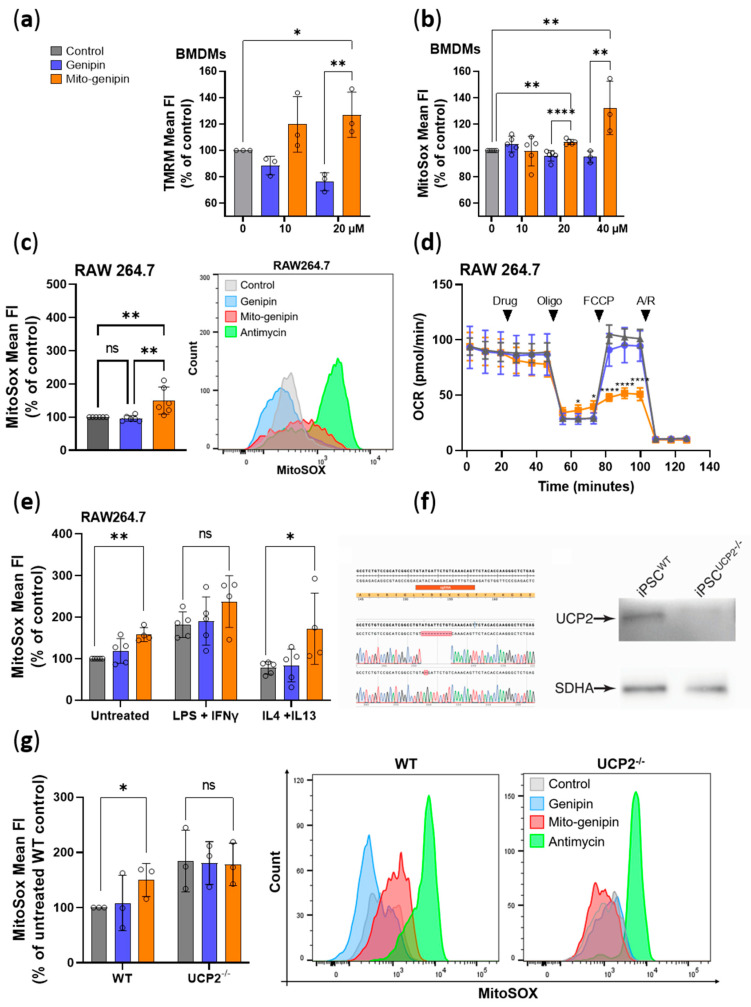
Effect of mito-genipin on mitochondrial functions. (**a**) Quantitative evaluation of the TMRM Mean Fluorescence Intensity of BMDMs untreated or treated with 10 μM and 20 μM mito-genipin or genipin for 60 s. Data were normalized on the mean Fluorescence Intensity of untreated BMDMs (n = 3 independent experiments). (**b**) Quantitative results of the MitoSox Mean Fluorescence Intensity (MFI) of BMDMs untreated or treated with 10 μM (n = 5), 20 μM (n = 5), and 40 μM (n = 3) mito-genipin or genipin for 30 min. (**c**) Quantitative results of the MFI of RAW 264.7 untreated or treated with 20 μM mito-genipin or genipin (n = 6) for 30 min. Representative histograms of MitoSOX MFI in RAW 264.7 cells under the indicated treatments. Data were normalized on the mean Fluorescence Intensity of untreated BMDMs. Data represent mean ± SD in an one-way ANOVA followed by Tukey’s multiple comparisons test. (**d**) Oxygen consumption rate (OCR) in RAW 264.7 cells was measured at preset time intervals following the preprogrammed addition of vehicle (DMSO), 20 μM mito-genipin or genipin and subsequent addition of mitochondrial respiration modulators. (**e**) Quantitative results of the MFI in polarized RAW 264.7 cells, untreated or treated with 20 μM mito-genipin or genipin for 24 h. Data represent mean ± SD in an unpaired Student’s *t*-test. (**f**) Sanger sequencing analysis of the two UCP2 alleles edited by CRISPR/Cas9, carrying 1-nt and 10-nt deletions, respectively. The orange box indicates the sgRNA target site on the reverse strand. Immunoblot analysis of total cell extracts from WT (iPSC^WT^) and UCP2 knockout (iPSC^UCP2−/−^) iPSCs using a polyclonal antibody specific for UCP2. An antibody against human SDHA was used as a loading control. (**g**) Quantitative results of the MFI of iPSCs WT and UCP^−/−^ untreated or treated with 20 μM mito-genipin or genipin (n = 3) for 30 min. Representative histograms of MitoSOX MFI in iPSCs WT and UCP^−/−^ cells under the indicated treatments. Data were normalized on the mean MFI untreated WT iPSCs. Data represent mean ± SD in an unpaired Student’s *t*-test. * *p* < 0.05; ** *p* < 0.01; **** *p* < 0.0001.

To investigate the effects of mito-genipin on mitochondrial function, we assessed mitochondrial membrane potential and ROS production in BMDMs. A rapid hyperpolarization was measured within 1 min after the addition of 20 μM mito-genipin (Figure 4a and Figure S2a for original data) and persisted for 15 min (Figure S2c–e). Importantly, the same concentration of genipin did not produce this effect. Consistent with observations that hyperpolarization causes the release of mitochondrial ROS [38], we detected a rapid increase in ROS production only after treatment with mito-genipin, not with genipin both in BMDMs (Figure 4b and Figure S2b for original data) and RAW 264.7 cells (Figure 4c and Figure S2f). This increase in ROS was detected as early as 30 min after treatment with 20 μM of mito-genipin. Next, we explored the effect of genipin and mito-genipin on respiration in intact RAW 264.7 cells (Figure 4d and Figure S2g), revealing that mito-genipin treatment affected mitochondrial function, as evidenced by an apparent reduction in uncoupled respiration. A possible explanation for this behavior is that the compound may induce some degree of toxicity towards electron transport chain components or increase sensitivity to oligomycin, (Figure 4d), similar to what has been observed with mitochondriotropic potassium channel inhibitors [25,39]. Altogether, these data indicate that already low doses of mito-genipin trigger rapid changes in mitochondrial membrane potential, respiration and ROS release in macrophages.

**Figure 5:**
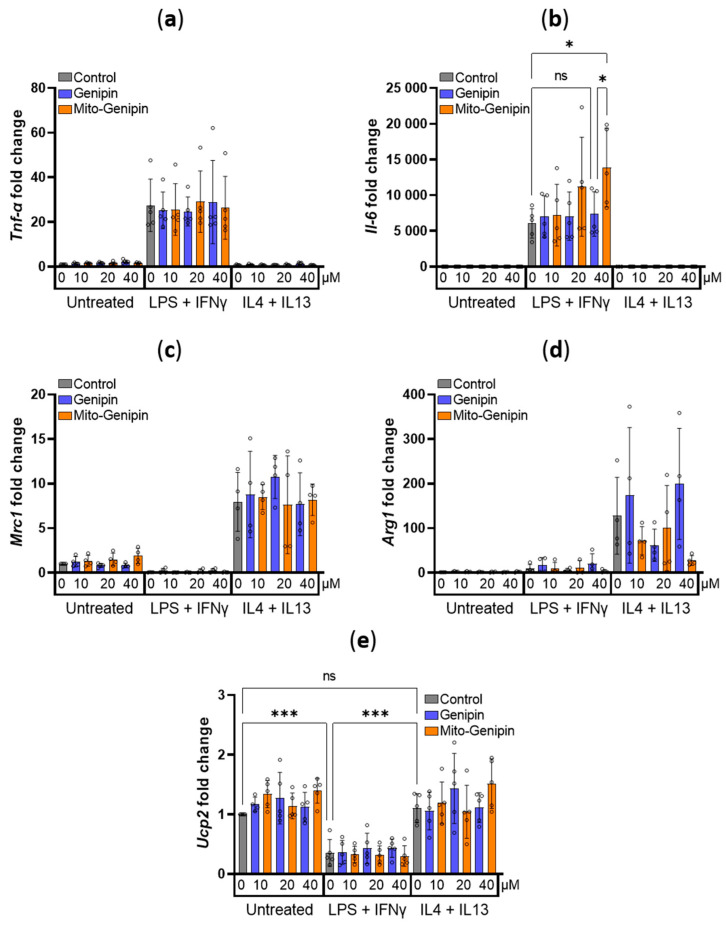
Effect of mito-genipin on macrophage polarization gene expression. Quantitative mRNA expression identified by RT-PCR of Tnf-α (**a**), Il-6 (**b**), Mrc1 (**c**), Arg1 (**d**) and Ucp2 (**e**) genes normalized to actin, and presented as fold change relative to untreated control in RAW 264.7 cells treated with 10, 20, and 40 mM genipin or mito-genipin and exposed to (LPS + IFN-γ), or (IL4 + IL13) for 24 h. Data represent ± SD from at least four independent experiments. * *p* < 0.05, *** *p* < 0.001, ns: not significant in one-way ANOVA followed by Tukey’s post hoc test.

The question arises whether mito-genipin induced ROS release depends on the presence of UCP2. To address this, we evaluated the effect of the drug on ROS release in M0, M1 and M2 polarized RAW 264.7 cells (Figure 4e and Figure S2h). Consistent with the strongly reduced expression of UCP2 in the M1 state (see Figure 5e and [40]) mito-genipin did not induce a significant increase in ROS release, in contrast to M0 and M2 polarized cells where UCP2 is highly expressed (Figure 4e). Moreover, the elevated ROS levels induced by mito-genipin in M0 and M2 cells persisted even 24 h after treatment (Figure 4e).

To further assess specificity of action, induced pluripotent stem cells (iPSC) stably knocked out for UCP2 (Figure 4f) were treated with mito-genipin. In the absence of UCP2, no increase in ROS level was observed (Figure 4g). In agreement with previous studies showing an enhanced ROS release in the absence of UCP2 [13], in UCP2 KO iPSC the basal ROS level was higher than in WT cells. Altogether, these data suggest that mito-genipin induces ROS release in a UCP2-dependent manner, indicating specificity of action.

### 3.5. Impact of Mito-Genipin on Selected Marker Genes of Macrophage Polarization

Given that mito-genipin rapidly hyperpolarizes mitochondria and elevates mitochondrial ROS, we asked whether it modulates the transcriptional program of classically (LPS + IFN-γ) or alternatively (IL-4 + IL-13) activated macrophages. We quantified mRNA for the pro-inflammatory markers *TNF-α* and *IL-6*, as well as the anti-inflammatory markers MRC1 and ARG1. Mito-genipin did not alter *Tnf-α* transcript level (Figure 5a). Notably, mito-genipin at a concentration of 40 μM induced a further substantial and significant increase in Il-6 expression in classically activated macrophages, which was not observed with the same dose of genipin (Figure 5b). By contrast, in alternatively activated macrophages, mito-genipin did not affect Mrc1 (Figure 5c); however, it showed a non-significant trend toward reduced Arg1 expression (Figure 5d). Because LPS + IFN-γ is known to downregulate Ucp2 transcripts [40], we also evaluated Ucp2 mRNA. Mito-genipin did not change Ucp2 expression in unstimulated or activated macrophages (Figure 5e). Collectively, these data indicate that mito-genipin selectively potentiates IL-6 in classically activated macrophages, with minimal impact on canonical anti-inflammatory markers.

### 3.6. Mito-Genipin Does Not Affect Metabolite Transport Activity Mediated by UCP2

**Figure 6:**
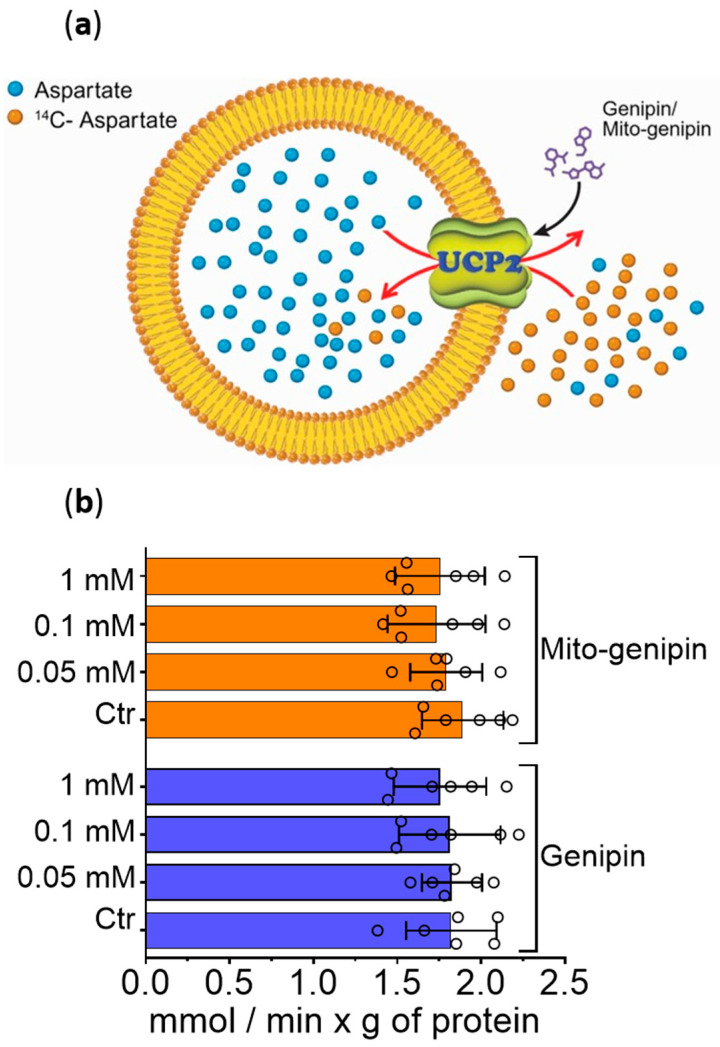
Mito-genipin does not inhibit the transport activity of recombinant human UCP2 reconstituted into liposomes. (**a**) Schematic representation of recombinant UCP-2 reconstitution into liposomes for aspartate transport assays. Proteoliposomes were preloaded with 20 mM aspartate, and transport was initiated by the addition of 1 mM [^14^C]-aspartate in the presence of varying concentrations (0.05–1 mM) of mito-genipin or genipin. Black arrow indicates that genipin/mito-genipin targets UCP2, which may transport aspartate in both directions (red arrows). (**b**) Recombinant human UCP2 was reconstituted into liposomes preloaded with 20 mM aspartate. Transport was initiated by the addition of 1 mM [^14^C]-aspartate and terminated after 1 min in the presence of various concentrations of genipin or mito-genipin. Data represents the mean ± SD of three independent experiments, each performed in two technical duplicates.

Finally, we aimed to investigate whether mito-genipin directly affects the metabolite transport activity of UCP2. To address this, recombinant human UCP2 was reconstituted into liposomes preloaded with 20 mM aspartate (Figure 6a). Transport activity was initiated by adding 1 mM [^14^C]-aspartate in the presence of various concentrations of mito-genipin or genipin. Neither compound altered significantly the UCP2-mediated aspartate transport (Figure 6b), indicating that mito-genipin does not directly affect the metabolite transport activity of UCP2.

## 4. Discussion

The use of genipin as an inhibitor of UCP2 originates from the study by Zhang et al. in 2006, who demonstrated its ability to block UCP2-mediated proton leak in pancreatic β-cells [1]. Since this initial finding, genipin has been widely adopted as a pharmacological tool to study UCP2 function in various cellular contexts, including macrophages [41]. Mechanistically, genipin is proposed to act at the level of the IMM, where it inhibits UCP2, leading to increased mitochondrial membrane potential, enhanced ATP production, and modulation of ROS levels, with downstream effects that vary depending on cell type [42,43,44,45]. However, the specificity of genipin toward UCP2 has been questioned, as it can also inhibit other UCP isoforms, raising concerns about off-target effects. Furthermore, UCP2 is deeply embedded within the IMM, which poses challenges for effective access by conventional inhibitors. Consequently, the clinical utility of genipin is further limited by its concentration-dependent behavior and strong crosslinking activity, both of which contribute to nonspecific effects [46].

To overcome these limitations, mitochondria-targeted derivatives of genipin represent a possibility to improve the specificity and potency of UCP2 inhibition. Therefore, we have developed mito-genipin. By strategically conjugating genipin to the lipophilic cation, TPP^+^, mito-genipin exploits the negative mitochondrial membrane potential to selectively accumulate within mitochondria [26]. Given the mitochondriotropic properties of TPP^+^ [47], it is reasonable to posit that mito-genipin would exert more potent UCP2 inhibition compared to the parent compound. As mentioned in the introduction, UCP2 is aberrantly expressed under various pathological conditions, including metabolic disorders, cancer, and inflammatory diseases [48,49]. Among UCPs, macrophages predominantly express UCP2 rather than UCP1 or UCP3 (https://www.proteinatlas.org/, accessed on 28 August 2025) [29,50], making them an ideal system for studying UCP2-specific effects without interference from other UCPs. In these cells, UCP2 plays a critical role in regulating mitochondrial ROS production, inflammatory signaling, and metabolic reprogramming during immune activation [17,51]. Therefore, we investigated the effects of mito-genipin on macrophage function.

Mito-genipin specifically accumulated in mitochondria and significantly altered the cytotoxic profile of macrophages at lower doses compared to genipin. Beyond cytotoxicity, mito-genipin induced rapid hyperpolarization of the IMM and increased mitochondrial ROS production at sublethal concentrations. Functionally, mito-genipin promoted selective upregulation of IL-6 during classical macrophage activation, with negligible anti-inflammatory markers in alternatively activated macrophages. These results are consistent with recent findings showing that genipin-mediated UCP2 inhibition in bacterially challenged alveolar macrophages restored mitochondrial ROS and consequently selective pro-inflammatory cytokines, including IL-6, without affecting TNF-α [20]. Notably, mito-genipin achieved similar effects at significantly lower concentrations, highlighting the enhanced potency in modulating specific pro-inflammatory responses. Although the precise mechanism of action of mito-genipin was not investigated here, our findings indicate that its effects are more likely mediated through modulation of mitochondrial function and ROS production rather than through direct regulation of UCP2 transcription or metabolite transport. As mentioned in the Section 1, UCP2 has been proposed to possess dual transport functions—facilitating both proton translocation and the export of C4 metabolites. The data presented here likely reflect modulation of UCP2’s proton-transporting activity specifically, rather than its role in substrate transport by mito-genipin. At low concentrations, genipin is proposed to decrease proton transport across UCP in the presence of long chain fatty acids, by binding to arginine residues located in the UCP protein [46]. It binds to UCP2 by interacting with the intermembrane space facing side of the protein, like GDP [46]. NMR studies highlighted that the nitroxide-labelled GDP binds inside the channel in proximity of transmembrane helices 1–4 [52]. The C1-linked hydroxyl group in genipin was identified as an active inhibitory group affecting UCP2 in pancreatic cells [33]. However, genipin (and likely mito-genipin) binds also to complex III of the respiratory chain, possibly contributing to the observed decrease in maximal respiration.

During the finalization of the present manuscript, a study was published that addressed a key limitation of genipin: its role as a natural crosslinker makes it unsuitable for direct in vivo use at higher concentrations. To overcome this, authors pre-polymerized genipin with glycine, generating Mito-G nanoparticles (~5 nm) that avoid nonspecific crosslinking while delivering UCP2 inhibition within pancreatic β-cells [53]. In contrast, our approach involved derivatizing genipin at the C10 position with a TPP^+^ moiety, resulting in mito-genipin, a mitochondria-targeted small molecule. Unlike Mito-G, mito-genipin may still engage in crosslinking reactions [54,55], as indicated by the formation of fluorescent cross-linking products at the level of mitochondria. While this might represent a potential limitation for systemic application at high concentrations, it also preserves the parent compound’s unique reactivity, allowing a chemically defined small molecule [32] to facilitate easier cellular and mitochondrial penetration. By contrast, the nanoparticle formulation employed by Mito-G might have different uptake kinetics and intracellular distribution [56]. Finally, it is worth noting that Mito-G was specifically engineered to accumulate within pancreatic β-cell mitochondria to suppress UCP2-driven autoinflammation in type 2 diabetes. By contrast, mito-genipin incorporating the TPP^+^ lipophilic cation may have broader applicability across different cell types and pathological contexts, where UCP2 is aberrantly expressed [57,58].

In summary, our study introduces mito-genipin as a novel mitochondriotropic putative UCP2 inhibitor, while not excluding potential pharmacological interactions with other mitochondrial proteins [46].

The limitations of the study include that the experiments presented here were conducted in vitro and ex vivo, specifically in murine macrophages. While these models provide useful insights for initial characterization, they do not fully recapitulate the complexity of in vivo systems. Consequently, the broader applicability of these findings to primary tissues and whole organisms remains to be established. In addition, the precise molecular mechanism through which mito-genipin exerts its effects was not addressed. Therefore, future in vivo studies will be useful to assess the therapeutic potential of mito-genipin, to evaluate its pharmacological behavior, and to further clarify both advantages and limitations of this mitochondria-targeted approach.

## 5. Conclusions

Mito-genipin emerges as a novel mitochondria-targeted putative UCP2 inhibitor that induces mitochondrial hyperpolarization and increases ROS, subsequently enhancing **IL-6** expression in classically activated macrophages. The compound acts by modulating mitochondrial function, rather than inhibiting UCP2 at the transcriptional level. These results highlight the utility of mitochondrial targeting to probe redox-dependent inflammatory signaling in macrophages and provide a foundation for further studies on UCP2 in immune regulation. The findings have potential relevance for pathological contexts characterized by dysregulated macrophage activation or chronic inflammation.

## Data Availability

All data related to this research are presented in the manuscript.

## Supplementary Materials

The following supporting information can be downloaded at: https://www.mdpi.com/article/10.3390/antiox14111281/s1, Figure S1: Relative fluorescence intensity (F/F0) of cells over time after treatment with genipin or mito-genipin (related to Figure 3); Figure S2: Effects of mito-genipin on mitochondrial functions (related to Figure 4).

## Abbreviations

The following abbreviations are used in this manuscript:

Arg1	Arginase 1
BMDMs	Bone marrow-derived macrophages
IFNγ	Interferon gamma
IL-4	Interleukin 4
IL-6	Interleukin 6
IL-13	Interleukin 13
IMM	Inner mitochondrial membrane
LPS	Lipopolysaccharide
Mrc1	Mannose receptor c-type 1
ROS	Reactive oxygen species
TFN-α	Tumor necrosis factor alpha
TPP^+^	Triphenylphosphonium
UCP2	Uncoupling protein 2
