# Supplementary material for: Mito-Genipin, a Novel Mitochondria-Targeted Genipin Derivative Modulates Oxidative Stress and Inflammation in Macrophages
Source: Antioxidants (Basel). 2025 Oct 25;14(11):1281. doi: 10.3390/antiox14111281 (PMC12649598; doi:10.3390/antiox14111281)
Supplement: Supplementary file 1 [file antioxidants-14-01281-s001.zip › antioxidants-3874047-supplementary.pdf]

# Mito-genipin, a Novel Mitochondria-Targeted Genipin Derivtive Modulates Oxidative Stress and Inflammation in Macrophages

**Beatrice Angi, Daria Di Molfetta, Diana Pendin, Giuseppe Antoniazzi, Carlo Alberto Flora, Francesco De Leonardis, Martina Buono, Giuseppe Fiermonte, Ildiko Szabo, Andrea Mattarei and Tatiana Varanita**

## Supplementary figures

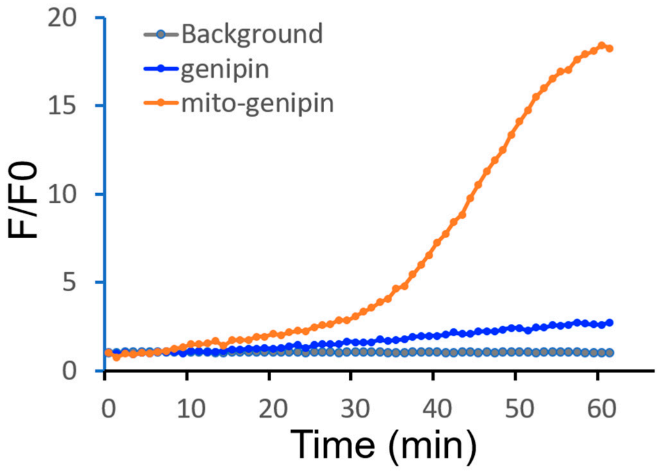


**Figure S1. Relative fluorescence intensity (F/F0) of cells over time after treatment with genipin or mito-genipin (related to Figure 3).** Relative fluorescence intensity (F/F0) of cells over time after treatment with 20 μM genipin (blue), mito-genipin (orange), or control (grey). Cells were imaged using fluorescence microscopy (ex: 555 nm, em: 620/52 nm) every 60 seconds for 1 hour. Background-subtracted fluorescence was normalized to initial values (F0).

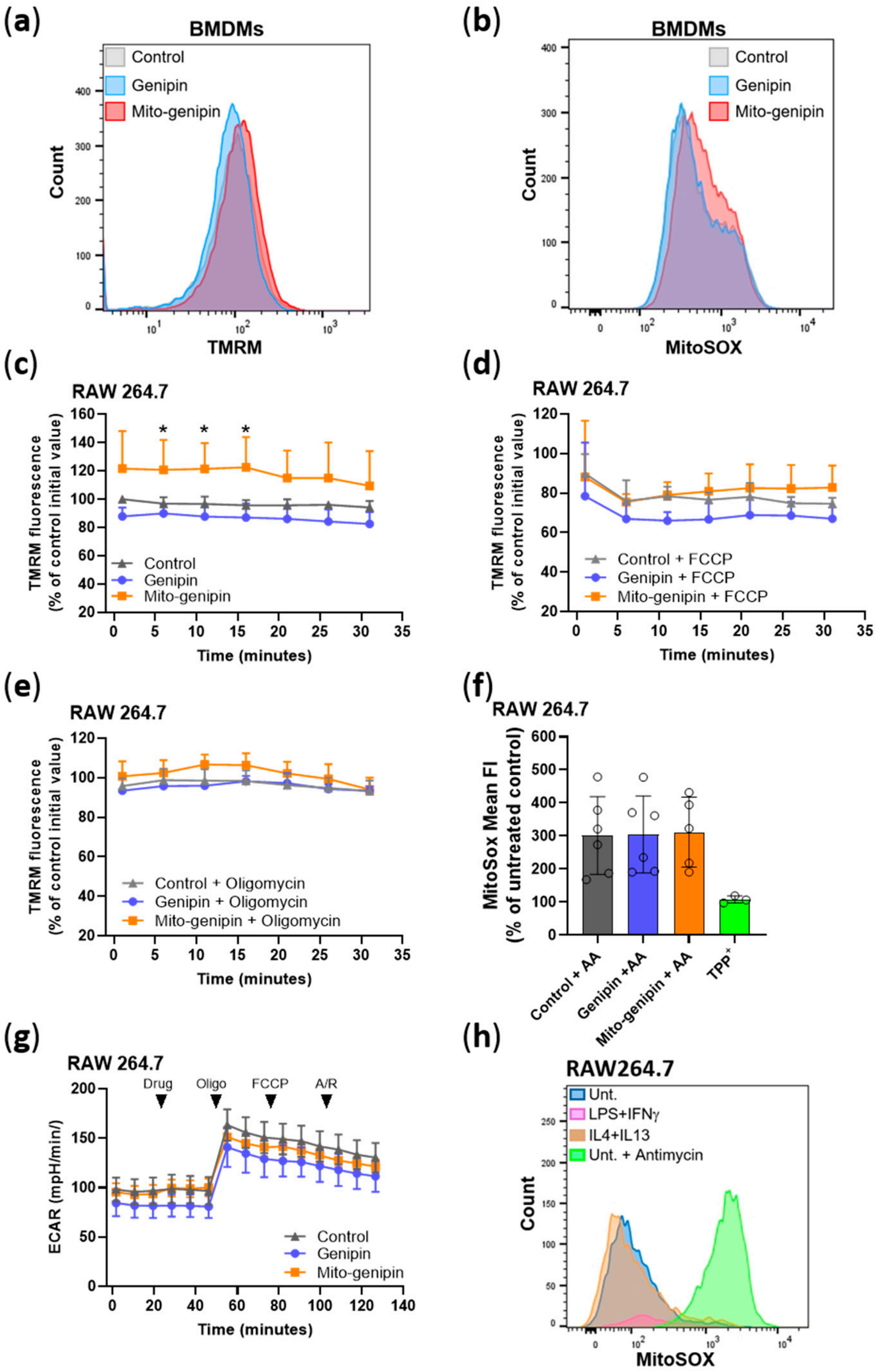


**Figure S2. Effects of mito-genipin on mitochondrial functions (related to Figure 4).** **(a)** Representative histograms of TMRM mean fluorescence intensity in BMDMs treated with 20 μM Genipin/Mito-genipin for 60 seconds. **(a)** Representative histograms of MitoSox mean fluorescence intensity in BMDMs treated with 20 μM Genipin/Mito-genipin for 30 minutes. **(c)** TMRM fluorescence kinetics of RAW264.7 cells, untreated (control) or treated with 20 μM genipin/mito-genipin. **(d)** TMRM fluorescence kinetics of RAW264.7 cells, untreated (control) or treated with 20 μM genipin/mito-genipin in the presence of the mitochondrial uncoupler FCCP (2 μM). **(e)** TMRM fluorescence kinetics of RAW264.7 cells, untreated (control) or treated with 20 μM genipin/mito-genipin in the presence of the ATP synthase inhibitor- oligomycin (2 μM). **(f)** Quantitative results of the MitoSox MFI of RAW264.7 treated with 20 μM $TPP^+$, 10 μM Antimycin A (AA), and 10 μM AA treated plus 20 μM mito-genipin or genipin for 30 minutes. **(g)** ECAR in RAW264.7 cells measured concurrently with OCR at preset time intervals following the preprogrammed addition of vehicle (DMSO), 20 μM mito-genipin or genipin, and subsequent addition of mitochondrial respiration modulators. This data complements the OCR measurements shown in Figure 4d. **(h)** Representative histograms showing MitoSOX MFI in RAW264.7 macrophages polarized for 24 hours with IFN-γ + LPS (M1), IL-4 + IL-13 (M2), or left untreated (M0).
